# Prevalence, predictors and risk perceptions of Hypertension among adults in Bududa Town Council, Eastern Uganda

**DOI:** 10.4314/ahs.v25i4.19

**Published:** 2025-12

**Authors:** James Kutosi, Abdul Walusansa, Abdulmujeeb Aremu Babatunde, Kharim Mwebaza Muluya, Ali Kudamba, Swaibu Zziwa, Irene Namasopo, Isaac Kuloba, Base Nabutsale, Francis Busiku, Mariam Namusoke, Cabral B Kibedi, Hussein Mukasa Kafeero

**Affiliations:** 1 Department Public and Community Health, Faculty of Health Sciences, Islamic University in Uganda, P. O. Box 7689, Kampala; 2 Department of Physiology, Habib Medical School, Faculty of Health Sciences, Islamic University in Uganda, P.O. Box 7689, Kampala; 3 Department of Biochemistry, Habib Medical School, Faculty of Health Sciences, Islamic University in Uganda, P.O. Box 7689, Kampala; 4 Department of Medical Microbiology, Habib Medical School, Faculty of Health Sciences, Islamic University in Uganda, P.O. Box 7689, Kampala; 5 Department of Biochemistry, Kabale University School of Medicine, P.O BOX, 317, Kabale; 6 Department of Biomedical and Mechatronics Engineering, Faculty of Engineering, Kyambogo University, P.O. Box 1, Kyambogo

**Keywords:** Hypertension, Prevalence, Adults, Risk factors

## Abstract

**Background:**

The global prevalence of hypertension has been on the increase in urban areas. Therefore, this study was aimed at establishing the prevalence and predictors of hypertension (HTN) among the adults of Bududa town council in order to inform the way forward on the prevention strategies.

**Methods:**

A mixed methods approach with a convergent parallel design was used. The quantitative arm involved 365 randomly selected participants, while the qualitative arm included 24 hypertensive patients. Quantitative data were analyzed at univariate, bivariate, and multivariate levels (p < 0.05), and thematic analysis was applied to the qualitative data.

**Results:**

The prevalence of HTN was 33.97% and was associated with age ≥ 40 years (AOR 0.2; 95% CI 0.13-0.36; p=0.000), high salt intake (AOR:0.4; 95%CI:0.24-0.78; P=0.005), sedentary life style (AOR: 0.5; 95% CI: 0.30-0.89; p=0.017), inappropriate source of information about the risk factors to hypertension, knowledge on primary prevention, antihypertensive side effects, stroke and poor adherence to antihypertensive.

**Conclusion:**

Hypertension remains a major public health issue in Bududa Town Council, closely linked to modifiable behaviors and limited risk awareness, including poor preventive knowledge, treatment non-adherence, and inadequate access to reliable health information.

## Introduction

Hypertension, defined as the Systolic Blood Pressure (SBP) values of 130mmHg or more and Diastolic Blood Pressure (DBP) values of more than 80mmHg^1^ is the most preventable cause of morbidity and mortality^2^. The global burden of hypertension exceeded 1.4 billion people and it is projected that over 1.6 billion people by 2025 will be hypertensive, should systolic blood pressure target of below 130mmHg become the uvisersal standard^3^. Over the past 25 years, for the period from 1990 to 2015, 3.5 billion people worldwide had systolic blood pressure between 110mmHg to 115mmHg which translated into 135.6 to 145.2 deaths per 100,000 persons, whilst those with SBP 140mmHg or higher were 874 million people with corresponding odds of 97.9 to 106.3 deaths per 100000 persons^4^. Unfortunately, about 88% of deaths due to hypertension have been recently reported from low- and middle-income countries (LMICs)^5^. The increase observed in the LMICs has been partly attributed to lack of awareness of the risk factors to HTN^6^.

In Sub-sarahan Africa (SSA), the prevalence of hypertension and pre-hypertension are high and they differ by population groups and are defined by occupation and degree of urbanization with overall prevalence at 25.9% and lowest among rural residents at 8.7%^7^.

In Uganda, Lunyera et al^8^. have reported a prevalence of 31.5% with central region posting the highest prevalence at 34.3% compared to western and eastern regions at 32.5% and 32.3% respectively. However, earlier studies reported a lower prevalence of HTN. For example, Kotwani et al^9^ reported a prevalence of 19.8% in Mbarara. Similarly, reported a prevalence of 27.2% in Mukono^10^. The differences in prevalence of HTN over time and by region can be attributable to the differences in the risk factors such as variations in level of salt intake, obesity, alcohol consumption, physical activity and diet, may explain some of the regional heterogeneity in hypertension prevalence^11^.

In Bududa hospital, evidence from Bududa District Health Management Information System (HIMS) indicates that there was a sharp increase in the number of cases diagnosed with hypertension from Bududa town from 278 cases in 2021 to 476 cases in 2022 making it the making it the 3rd leading cause of mortality and 8^th^ cause of morbidity among people aged 5years and above as revealed by the financial year 2021/2022 health report. However, the predictors of increasing HTN in Bududa town council are not known.

Elsewhere, the predictors of hypertension have been highlighted including:- First, gender differences with men particularly those who are married and with the propensity of high salt intake being at a higher risk than women, high body mass index^12^-^14^. Second, smoking 15. Third, low or no formal education 15. Fourth, obesity, alcohol consumption, physical inactivity and unhealthy diet^11^. Lastly age ≥ 18 years 16. In addition, low awareness of the risk factors to HTN and poor perceptions of being at risk of developing HTN have been highlighted elsewhere as predictors of HTN^6^.

Therefore, this study sought to determine the prevalence, risk factors and risk perceptions associated hypertension among adults of Bududa town council, Bududa district in order to inform the way forward in designing the interventions to mitigate the escalating cases of HTN in Bududa town council.

## Methods

### Study setting, design, sample size and population

The study was conducted in Bududa town, Bududa district in Bugisu sub-region in Eastern region of Uganda, about 38 kilometers southeast of Mbale district headquarter. The town council has 1,548 households with a projected population 20,532 persons for year the 2023/24. The study included participants aged 18 years and above who were mobilized to one place as designated by local authorities as a center place for social gathering. All adults of Bududa town council aged 18 and above with a sound mind, willing to participate upon filling and signing an informed consent form were eligible to participate in the study.

The study employed a mixed methods research approach undertaking a convergent parallel design. The quantitative methods were used to assess the prevalence and risk factors associated with hypertension whilst qualitative methods were used to explore the risk perception associated with hypertension among the adults of Bududa town council. Sample size of 365 participants was determined using Kish Leslie formula of 1965 and a total of 24 patients with hypertension were recruited into focused group discussion and this number was attained by saturation.

### Sampling techniques

A simple random sampling method was used to select 365 participants who were randomly selected from a sample frame of persons aged 18 and above compiled from Bududa town council. The sample list selected by simple random sampling used excel Micro software package with the help of RAND command to cause even distribution of participants in the excel spread sheet. The sample list was later arranged by village and was given to mobilizers from respective villages who were residents and reached out to randomly selected participants to participate in the study. Those who were absent were followed by mobilizers from their respective villages/cells through either phone call or home visit. Those who declined to participant in the study were replaced by the participants who were next after the 365 number in the excel sheet by simple random sampling.

The purposive sampling technique was applied to select participants to participate in the focused group discussions who were found hypertensive at the time of carrying out the quantitative survey. The hypertensive participants who were listed during the quantitative study and were informed using phone call to participate in focus group discussions (FGDs). The participants for FGD were selected based on age category, gender, location with each group having 6 participants. According to Johnson and Christensen (2004) 6-12 participants but a small group of 6-8 is appropriate and easy to control during discussion. The homogenous groups based on the above characteristics were used.

### Data collection tools and procedure

The research questionnaire from the World Health Organization (WHO) was used. A structured questionnaire was used to collect data for quantitative study and semi-structured interview guide was used for the qualitative data. The research tool had three sections: socio-demographic data, prevalence and risk factors. The questionnaire was written in the English language and two research assistants were used in data collection. The questionnaire was interpreted to participants from English to Lumasaba language. The structured questionnaire was used because the format is familiar to most respondents, they are straight forward to analyze and simple to administer. The interviewer administration of the questionnaire was used to ensure the completeness and accuracy of the research tool.

The FGDs interview were conducted to groups which were selected purposively upon listing hypertensive persons during quantitative data collection. The interview guide for the FDGs was discussed with the supervisor and was pretested among community members from Nangako town council. The guide for FGD had the following question: What are the diseases that are common in your area? Is hypertension a problem in your community? What are the reasons for causing hypertension? What can be done to prevent hypertension? Describe the problems you experience in your body due to daily use of antihypertensive? What are the complications of the hypertension? What treatment plan has your doctor recommended for you? Are you able to follow this treatment daily? If not, what plan do you find hard to follow? Do you feel that the treatment plan is helping control your disease? If not, why not?

### Study variable and measurement

The dependent variable of the study was prevalence of hypertension of which hypertension was defined as an average measured systolic pressure of ≥140mmHg and diastolic blood pressure ≥90mmHg or by history. The overall prevalence was recorded as binary categorical outcome as Yes with a true logical expression of one (1) and no with false logical expression of zero (0). This approach was used by a similar study conducted in Uganda (Nuwaha & Musinguzi, 2013) while the independent variables were; risk factors (age, sex, education, occupation, income, tobacco use, alcohol consumption, body mass index and physical activity) and risk perceptions which emerged with five thematic areas: - prevention and control, side effects of antihypertensive, complications and treatment adherence.

The study variables were measured in reference to (10) whereby; Physical activity: participants were asked the number of times they got involved in physical activity such as walking, riding, manual work, exercises and sports. Those who reported at least 3 times in the week were categorized as frequent and those less than 3 times as infrequent. Age: The age of participants was measured as a continuous variable but during analysis was categorized into three categories of 18-39 (youth & young adults), 40 and above as middle-aged adults and, the elderly. This was because the sample size of 365 participants if over categorized will lose power. Education status: those who had completed primary and no schooling were classified as Primary & below while those who had completed secondary, high school, university/college and post graduate degree as secondary and above. Occupation: was classified as formal meaning employed by government or non-government organization (NGO) while informal self-employed, student, retirees extra. Income: income was classified based on World Bank Country categories by FY2023/24 as extreme poverty/low-income earners being people who earned less than $1135 per year an equivalent of shillings 4,199,500 Uganda currency and low-middle income earner and above being those who earned above $1136. Alcohol use: Was categorized into two based on the measure of alcohol usage among participants that is; none & current light drinkers and moderate-heavier drinkers. Current light drinkers: At least 12 drinks in the past year but 3 drinks or fewer per week, on average over the past year. Current moderate drinker: More than 3 drinks but no more than 7 drinks per week for women and more than 3 drinks but no more than 14 drinks per week for men, on average over the past year. Current heavier drinker: More than 7 drinks per week for women; more than 14 drinks per week for men, on average over the past year^17^. Body mass index (BMI): This was measured by measuring weight in kilograms divided by height in meters squared. It was categorized into two; Normal (BMI less than 25) and overweight/obese (BMI above 25 for analysis purposes. Tobacco use; Three categories were used; regular smoker (daily smokers), former smoker (adult who has smoked at least 100 cigarettes in his or her lifetime but who had quit smoking at the time of interview) and never smoker: An adult who has never smoked, or who has smoked less than 100 cigarettes in his or her lifetime^18^. Vegetable and fruit consumption; the consumption of vegetables and fruits was measured by asking participants how many days they consumed fruits or vegetables in a week. Those who reported 7 days of smocking in a week were classified as frequent and those who reported less than 7 days as infrequent. Finally, those who had one serving or more in the day were classified sufficient users and less or none as insufficient users

### Data quality control measure and analysis

To ensure quality assurance and control measures, the data collection tool was adopted from WHO and was pre-tested, trained and monitored data collectors, recorded data collection processes, performed data cleaning, coding and verification of entries in Excel sheet was done. Thereafter data were analyzed with the help of STATA version 14.0 using Logistic regression model and were presented using appropriate frequency tables and percentages. The data underwent three phases that is uni-variate analysis to compute for proportions and frequencies. At Bivariate analysis cross tabulation between the dependent (HTN prevalence) and associated factors was performed at time to determine their level of significance using chi-square, p-value at<0.05 and 95% confidence interval. To select variable for multivariate analysis a P-value of <0.2 cutoff was used. This approach was used in asimilar study in Uganda^19^.

After focused group discussion, all conversations were transcribed verbatim into Lumasaba/Lugisu language while listening to the audiotaped by a trusted research assistant and the researcher for quality control in qualitative research. The transcripts were compared for similarity in content and after thorough checks, the transcripts were translated into English. Similar quality control measures were followed to ensure credibility and transferability of results. For the qualitative analysis, thematic analysis was done. The six steps by Braun and Clarke 2006 were applied that is to; become familiar with the data, generate initial codes, search for the themes, review the themes, and define the themes and write-up the theme.

## Ethical considerations

Since the host institution for all the authors did not have an approved Research and Ethics Committee (REC) by the National Council for Higher Education (NCHE), ethical approval was sought and obtained from the Uganda Christian University-Research and ethics committee (UCUREC), approval number UG-REC-026 of application number UCUREC-2023-813. The research team explained the consent form to all the participants and signed accordingly. Participant's rights to privacy and confidentiality were kept throughout the study as laid down in the Helsinki declaration.

## Results

### The socio-demographic characteristics of the study participants

A total of 365 participants were recruited in the study of which 214 (58.6%) were females. Majority were aged ≥ 40 years (200, 54.8%) with a mean age ± SD of 43.3 ±17.5, In addition, majority (287, 78.6%) had completed primary level of education, married (221, 60.5%), with Gisu ethnicity (355, 97.3%), informally employed with majority (346, 94.8%) being low-income (Table 1).

**Table 1:** The socio-demographic characteristics of the study participants

Variables	Category	Frequency	Percentage
**Sex**	Female	214	58.6%
	Male	151	41.4%
**Duration of schooling**	Mean & SD time spent at school among		
	participants was 7.3±4.7 years		
**Age**	Below 40(Youth/Young adults)	165	45.2%
	Above 40(Middle/elderly aged)	200	54.8%
	**Mean age &SD of 43.3±17.5**		
**Education**	Primary & No school	287	78.6%
	Secondary & above	78	21.4%
**Marital status**	Married	221	60.5%
	Single	144	39.5%
**Ethnicity**	Bagisu	355	97.3%
	Others	10	2.7%
**Occupation**	Formal	53	14.5%
	Informal	312	85.5%
**Income**	Extreme poverty/low-income earners	346	94.8%
	Low-middle & above earner	19	5.2%

**Abbreviation: SD=Standard deviation**

### The prevalence of hypertension among participants

Overall, the prevalence of hypertension among the participants in the study area was 33.97%. The prevalence of hypertension was significantly different among study participants by age (chi-square, 43.68; 95%CI 0.13-0.34; p=0.000) others who; ever had their BP measured by HWs (chi-square, 40.75; 95%CI 0.14-0.37;p=0.000), ever been told by HWs that their BP is raised (chi-square, 119.58; 95%CI 0.03-0.10; p=0.000), told in the past 12months that they have HTN (chi-square,78.29; 95%cl 0.06-0.17; p=0.000), requested HWs to check their BP status (chisquare, 43.78; 95%CI 0.13-0.34; p=0.000), had ever thought of getting automatic BP machine to self-check their BP at home (chi-square, 58.19; 95%bCI 0.05-0.19; p=0.000), were able to read BP machine results at home (chi-square, 19.66; 95%CI 0.06-0.38; p=0.000), were able to read and interpret BP results when recorded for them (chi-square, 12.97; 95%CI 0.09-0.45; p=0.001), thought that knowing raised BP helps them take their medicines well (chi-square,30.78; 95%CI 0.18-0.45; p=0.000) and current tobacco users(chi-square,5.95; 95%CI 0.18-0.83; p=0.015) (Table 2)

**Table 2:** The prevalence of hypertension and its differences by other factors

Variables	Category	Hypertension		X^2^	95%cl
				
N=365		Yes (%)	No (%)		
	HTN	124(33.97)	241(66.03)		
	prevalence				
Sex	Female	67(31.31)	147(68.69)	Ref	1
	Male	57(37.75)	94(62.25)	1.63	0.48 to 1.16
Age	Above 40	97(48.50)	103(51.50)	43.68	0.13 to 0.34
	Below 40	27(16.36)	138(83.64)	Ref	1
Classification of HTN by partns	HTN	124(33.97)		Ref	1
	Pre-HTN		114(31.23)	0.202	-9.1 to 14.4
	Norm-HTN		127(34.79)	0.0185	-10.8 to 12.4
Ever had BP measured by HWs	Yes	88(50.29)	87(49.71)	40.75	0.14 to 0.37
	No	36(18.95)	154(81.05)	Ref	1
Told by HWs that has	Yes	76(80.0)	19(20.0)	119.58	0.03 to 0.10
HTN	No	48(17.84)	221(82.16)	Ref	1
Told in past 12months about BP?	Yes	64(73.56)	23(26.44)	78.29	0.056 to 0.17
	No	59(21.30)	218(78.70)	Ref	1
Requested HWs to check your BP	Yes	68(58.12)	49(41.88)	43.78	0.13 to 0.34
	No	56(22.58)	192(77.42)	Ref	1
Has a plan to get an automatic BP machine	Yes	46(77.97)	13(22.03)	58.19	0.05 to 0.19
	No	78(25.49)	228(74.51)	Ref	1
Able to read BP results	Yes	20(74.07)	7(25.93)	19.66	0.06 to 0.38
	No	104(30.77)	234(69.23)	Ref	1
Able to read and interpret BP results	Yes	17(68.00)	8(32.00)	12.97	0.09 to 0.52
	No	107(31.47)	233(68.53)	Ref	1
Knowing BP results helps adherence	Yes	84(48.28)	90(51.72)	30.78	0.18 to 0.45
	No	40(20.94)	151(79.06)	Ref	1
Currently smoke	Yes	16(55.(7)	13(44.83)	5.95	0.18 to 0.83
	No	108(32.14)	228(67.86)	Ref	1
Consumes alcoholic drinks	Yes	70(38.46)	112(61.54)	3.27	0.43 to 1.03
	No	54(29.51)	129(70.49)	Ref	1
Involged MVPA like digging or loads lifting	Yes	94(32.19)	198(67.81)	2.02	0.87 to 2.49
	No	30(41.10)	43(58.90)	Ref	
Consumption of fruits in a week	Frequent	9(32.14)	19(67.86)	0.05	0.48 to 2.49
	Infrequent	115(34.12)	222(65.88)	Ref	1
Consumption of vegetables in a week	Frequent	23(29.11)	56(70.89)	1.08	0.77 to 2.29
	Infrequent	101(35.31)	185(64.69)	Ref.	1

Abbreviations: HTN=Hypertension, BP=Blood pressure, HWs= Health workers *p value significant at p<0.05 and 95% confidence intervals

### The risk factors associated with hypertension among adults of Bududa town council, Bududa district

We performed a bivariate analysis of socio-demographic characteristics associated with the risk of HTN. The age ≥40 years (COR 0.2; 95% CI 0.13-0.34; p=0.000), education level ≥ secondary (COR 1.8; 95% CI 1.01-3.17; p=0.045) were significantly associated with HTN (Supplemental Table S1). Furthermore, smoking (COR:0.4; 95%CI: 0.18-0.83; p=0.015) (Supplemental Table S2), alcohol consumption (COR:1.9; 95%CI: 1.059-3.670; p=0′032) (Supplemental Table S3), dietary intake of excess salt (COR: 0.4; 95% CI: 0.25-0.73; p=0.002) (Supplemental Table S4) and frequent involvement in fitness or recreational activity for ≥ 10 minutes (COR: 0.5; 95%CI: 0.32-0.78; p=0.002) compared to who had (Supplemental Table S5).

We also performed a multivariate analysis for variables that qualified for the analysis. The following factors were significantly associated with hypertension among the adults of Bududa town council. First age: - being aged ≥ 40 years (AOR 0.2; 95% CI 0.13-0.36; p=0.000). Therefore being 40 years and above increased the risk of hypertension by 20% compared to those participants who were < 40 years (Table 3),

**Table 3:** Multivariate analysis of the socio-demographic characteristics as risk factor associated with hypertension

Variable	Hypertension outcomes		COR (95%CI)	PV	AOR (95%Cl)	P-V

	Yes (%)	No (%)				
**Sex**						
Male	57(37.75%)	94(62.25%)	0.7(0.48-1.16)	0.201	0.8(0.52-1.32)	0.435
Female	67(31.31%)	147(68.69%)	Ref		Ref	
**Age**						
Above 40 years	97(48.50%)	103(51.50%)	0.2(0.13-0.34)	0.000*	0.2(0.13-0.36)	0.000*
Below 40 years	27(16.36%)	138(83.64%)	Ref		Ref	
**Education Level**						
Secondary & above	19(24.36%)	59(75.64%)	1.8(1.01-3.17)	0.045*	1.2(0.67-2.32)	0.476
Primary & No school	105(36.59%)	182(63.41%)	Ref		Ref	
**Marital status**						
Single	41(28.47%)	103(71.53%)	1.5(0.96-2.38)	0.074	1.3(0.84-2.19)	0.218
Married	83(37.56%)	138(62.44%)	Ref		Ref	

Abbreviations: COR=crude odds ratio, AOR=adjusted odds ratio

* p value significant at p<0.05 and 95% confidence intervals

Second, daily tobacco smocking in the past (AOR:0.4; 95%CI:0.18-0.89; p=0.025). Hence daily tobacco smocking increased the risk of hypertension by 40% (Table 4).

**Table 4:** Multivariate analysis of Tobacco use as a risk factor associated with hypertension

Variable	Response	Hypertension outcomes		COR	P-V	AOR (95%Cl)	P-value

		Yes (%)	No (%)				
**Current smokers**	**Yes**	16(55.17%)	13(44.83%)	0.4(0.18-0.83)	0.015*	0.6(0.27-1.39)	0.241
	**No**	108(32.14%)	228(67.86%)	Ref		Ref	
**Daily smokers**	Yes	11(57.89%)	8(42.11%)	0.3(0.14-0.90)	0.029*	0.5(0.10-3.09)	0.491
	No	5(50.50%)	5(50.50%)	Ref		Ref	
**Tried to stop smoking in past 12 months**	Yes	5(38.46%)	8(61.54%)	0.3(0.06-1.32)	0.109	5.6(0.89-33.41)	0.067
							
	No	11(68.75%)	5(31.25%)	Ref		Ref	
**Advised to quit smoke in past 12months**	Yes	1(16.67%)	5(83.33%)	9.4(0.93-94.65)	0.058	6.5(0.59-70.38)	0.126
							
							
	No	15(65.22%)	8(34.78%)	Ref		Ref	
**Smoked daily in the past**	Yes	20(62.50%)	12(37.50%)	0.3(0.13-0.58)	0.001*	0.4(0.18-0.89)	0.025*
							
	No	104(31.23%)	229(68.77%)	Ref		Ref	
**Currently use smokeless**	Yes	9(52.94%)	8(47.06%)	0.4(0.16-1.17)	0.099	0.7(0.23-1.89)	0.441
							
	No	115(33.05%)	233(66.95%)	Ref		Ref	
**Exposed to smoke**	Yes	45(40.91%)	65(59.09%)	0.6(0.41-1.03)	0.067	0.7(0.45-1.23)	0.238
	No	79(30.98%)	176(69.02%)	Ref		Ref	

Abbreviations: COR=crude odds ratio, AOR=adjusted odds ratio

* p value significant at p<0.05 and 95% confidence intervals

Third, stopping drinking alcohol due to health reasons (COR:2.1; 95%CI: 1.10-4.05; p=0.025). thus, the risk of developing hypertension was 2.1 times higher among those who were drinking alcohol but medically told to stop for health reasons (Table 5).

**Table 5:** Multivariate analysis of alcohol consumption as risk factor associated with hypertension

Variable		Hypertension outcomes		COR (95%CI)	P-Value	AOR (95%Cl)	P-Value

		Yes (%)	No (%)				
**Ever consumed alcoholic drinks**	Yes	70(38.46%)	112(61.54%)	0.7(0.433-1.036)	0.072	0.7(0.47-1.20)	0.235
						
	No	54(29.51%)	129(70.49%)	Ref		Ref	
**Stopped drinking due to health reasons**	Yes	23(29.49%)	55(70.51%)	1.9(1.059-3.670)	0.032*	2.1(1.10-4.05)	0.025*
						
	No	47(45.19%)	57(54.81%)	Ref		Ref	

Abbreviation: COR=crude odds ratio, AOR=adjusted odds ratio, MHD= Moderate/heavier drinkers: NLD= None/light drinkers

* p value significant at p<0.05 and 95% confidence intervals

Fourth, much salt consumption (AOR:0.4; 95%CI:0.24-0.78; P=0.005). Hence, high salt intake increased the risk of hypertension by 40% compared to those who took normal salt levels (Table 6).

**Table 6:** Multivariate analysis of dietary as risk factor associated with hypertension

Variable	Hypertension outcomes		COR (95%CI)	P-Value	AOR (95%Cl)	P-Value

	Yes (%)	No (%)				
**No. servings of fruits eaten in a week**						
					
Sufficient	114(32.85)	233(67.15)	Ref		Ref	
Insufficient	10(55.56)	8(44.44)	0.4(0.15-1.02)	0.055	0.5(0.21-1.47)	0.230
**Eats processed food high in salt?**						
					
Yes	64((38.32)	103(61.68)	0.7(0.45-1.08)	0.108	0.6(0.41-1.05)	0.077
No	60(30.30)	138(69.70)	Ref		Ref	
**Amount of salt consumed**						
					
Much	33(50.77%)	32(49.23%)	0.4(0.246-0.732)	0.002*	0.4(0.24-0.78)	0.005*
Normal	91(30.43%)	208(69.57%)	Ref		Ref	

Abbreviation: COR=crude odds ratio, AOR=adjusted odds ratio

* p value significant at p<0.05 and 95% confidence intervals

And finally, frequent involvement in sports, fitness or recreational activity) for ≥ 10 minutes (COR: 0.5; 95%CI: 0.32-0.82; p=0.005). This reduced the risk by 50% compared to those without frequent involvement in sports, fitness or recreational activity (Table 7).

**Table 7:** Multivariate analysis of Physical activity as risk factor associated with hypertension

Variable	Hypertension outcomes		COR (95%CI)	P-Value	Adjusted odds ratio (95%Cl)	P-value

	Yes (%)	No (%)				
**Involved in VPA by lifting heavy load, digging & construction works**						
**Yes**	29(40.85)	42(59.15)	Ref	0.174	Ref	0.118
**No**	95(32.31)	199(67.69)	0.7(0.41-1.18)		0.6(0.36-1.12)	
**Involved in VPA by walking or bicycle for ≥10min**						
Yes	103(32.00%)	212(67.30%)	Ref		Ref	
No	21(42.00%)	29(58.00%)	0.7(0.36-1.230	0.199	0.8(0.41-1.52)	0.486
**No. of days involved in VPA by walking or bicycle for ≥10min**						
Infrequent	64(29.09%)	156(70.91%)	0.6(0.36-0.97)		0.5(0.30-0.89)	
Frequent	39(41.05%)	56(58.95%)	Ref	0.039*	Ref	0.017*
**Involved in VPA by sports, fitness or recreational activity for ≥10min**						
Yes	61(27.73%)	159(72.27%)	Ref		Ref	
No	63(43.45%)	82(56.55%)	0.5(0.32-0.78)	0.002*	0.5(0.32-0.82)	0.005*
**BMI Overweight-Obese Normal**						
						
	38(40.86%)	55(59.14%)	0.7(0.411-1.088)		0.6(0.35-1.02)	0.062
	86(31.62%)	186(68.38%)	Ref	0.105	Ref	

Abbreviations: - COR=crude odds ratio, AOR=adjusted odds ratio, VPA: Vigorous physical activity, BMI: Body mass index

* p value significant at p<0.05 and 95% confidence intervals

### The results of the risk perceptions associated with hypertension among adults of adults of Bududa town council, Bududa district

Twenty-four hypertensive patients were recruited in this study. The six major themes were developed via thematic content analysis, this included common disease in the community, causes of pressure, prevention and control of pressure, side effects of antihypertensive, complications and treatment.

#### Theme 1: Common disease in the community

Two subthemes were identified in the context of common diseases affecting the community, these were non-communicable and communicable diseases.

### Non-communicable diseases

All the focused group discussions mentioned symptoms like paralysis of legs or hands which are symptoms of non-communicable diseases like pressure and diabetes.

*“Nowadays doctor we have pressure, diabetes because even my brother has pressure” FGD1,2,3 &4 “I have seen a child of 26 years in my area with pressure FGD2 & ulcer, this is too much*!!!” FGD2

### Communicable diseases

Participants revealed that the malaria, cough, skin itching and diarrhea were the diseases that were disturbing the community.

*“I have seen Malaria, diarrhea and cough” FGD2 “Our neighbors have children and elderly person with skin itching”* FGD2

#### Theme 2: causes of pressure/hypertension

Three subthemes were identified in the context of risk factors associated by hypertension.

### Unknown cause

Participants said that the cause of hypertension was not known to them because they realized that the hypertension was even affecting young people.

*“Pressure is just problem of its own because children are having pressure to, which problems are they having?”* FGD4. *“I don't really understand the cause of pressure but I think my pressure came as a result of stressful thoughts when my husband decided to marry another wife.”* FGD1

### Misconceptions on the causes of pressure

Although minority of the participants reported misconceptions like witch craft as cause of pressure, this information would be underestimated because the source of information was a religious leader whom many can be misled.

*“My pastor told me that I have stepped on witchcraft which caused me paralysis”* FGD1

### Persistent stressors

The participants revealed that they get stress from many ranging from paying school fees, feeding children, domestic issues, conflicts from neighbors but poverty was major stressor.

*“I don't sleep because of stress, as woman I have given birth to children but children misbehavior leading to imprisonment of them, this have caused me stressful thoughts”* FGD4 *“Stress due to problem of finances but above all we see poverty as cause of pressure.”* FGD1 &2

### Consumption of unhealthy diet

Participant said pressure is on an increase due to unhealthy eating habits they have copied from white/westerners. They revealed frying of food is a common practice in the community and they confirmed that even them they cannot eat un-fried food because they are tasteless.

*“I see in the past pressure was not common becausefryingfoods was not common.”* FGD1 *“I used to eat much salt before knowing that I have pressure.”* FGD2

#### Theme 3: prevention and control of pressure

Three subthemes emerged during content analysis on prevention and control of pressure. They included adaptation of good stress coping strategies, promotion of health seeking behaviors from experts and health lifestyle adaptation.

### Adaptation of good stress coping strategies

The participants disclosed that if they are to prevent and control pressure stress should be avoid but others expressed fear that it's difficult to avoid stress.

*“I have lost six children but you cannot tell because I have prepared my mind that in life anything can happen!”* FGD1. *“To me prayers helped me to reduce the pressure”* FGD1&2

Promotion of health seeking behaviors from experts The participants said that they take long to decide to seek health advice from experts and they often get wrong information from friends, spiritual leaders and herbalists.

*“Minimizing usage of private health setting because they are money oriented because you go with different prescription, they give you a different one.”* FGD1. “What I know health workers should guide us on what to do prevent pressure.” FGD4 *“Creation of health promotion groups in community to health educate us.”* FGD1

health lifestyle adaptation

The participant realized a need to change unhealthy life style behaviors that affect their blood pressure outcomes. Like doing physical exercises, reducing salt intake, restricting fats intake and sugars.

*“Reducing salt consumption, fats, sugar and exercises.”* FGD 3 *Theme 4: side effect of antihypertensive*

Two subthemes were identified during content analysis. Participants reported cardiovascular and neurologic side effects. However, the exact drugs that caused those side effects were not known to them.

### Cardiovascular side effects

The participants revealed that they feel headache and sweats after swallowing their antihypertensive medicines.

*“When I swallow, I feel headache.” FGD 1, 2, 3 &4. “I feel sweat episodes.”* FGD 1

### Neurologic side effects

The same participants revealed that before starting anti-hypertensive they were not feeling burning sensations but the symptoms of burning sensations started after some time on treatment.

*“I feel burning sensations in the feet and hands.”* FGD3

#### Theme 5: complication of hypertension

Two subthemes were identified during content of complication of hypertension, this included Neuro-cardiovascular and endocrine complications.

### Neuro-cardiovascular complication

The participant reported having numbness or paralysis of legs and hands, neck pain and blurred vision which may progress to blindness.

*“I feel hungry all the time because of pressure, oh my face looks like tory due to swelling.”* FGD 4

*“Numbness of the body, stroke and neck pain.”* FGD 1, 2 & 3

### Endocrine complications

The participants said hypertension and diabetes are like brothers and sisters because when you get the likelihood of getting the other is high.

*“I was first diagnosed with hypertension later I was told that I have diabetes again.”* FGD3

#### Theme 6: Treatment Adherence

Two subthemes were identified on treatment adherence as either being good or bad.

### Good treatment adherence

These are participants who swallowed their medicine as instructed by health providers.

*“I used to miss swallowing my medicines but when I had that pressure cause stroke I now swallow well”* FGD 2

*“They stopped me from medicines because my pressure is normal since December 2023.”* FGD 1

### Poor treatment adherence

Some participants were not swallowing their medicines as instructed by health provider and they are at potential risk of developing complication.

*“I am no longer taking because I don't get medicines in the hospital.”* FGD1

“I don't swallow my medicines regularly.” FGD 4

## Discussion

The findings of the study indicated that 33.97% of adults in the study area were hypertensive, presenting a significant perceived health threat as outlined in the Health Belief Model (HBM). This prevalence is notably higher than that reported in several other populations, including 23.2% among Chinese adults aged 18 and over^16^, 25.9% among urban populations in Sub-Saharan Africa 7, and various regions within Uganda. For instance, earlier studies recorded hypertension rates of 19.8% in Mbarara^9^, 27.2% in Mukono and Buikwe 10, and 20.8% in Rakai district 20. The elevated rate observed in this study may be linked to limited engagement with health-promoting activities at the primordial and primary levels of prevention.

The study found that hypertension prevalence differed by sex, with 37.75% of males and 31.31% of females affected. These results are consistent with findings from other regions, such as the United States, where hypertension was reported at 51.7% in men and 42.8% in women^13^; in India, 12.5% of men and 11.3% of women 21; and in Uganda, with 28.3% of men and 25.2% of women affected 7. Within the framework of the Health Belief Model (HBM), sex may be viewed as a factor that influences perceived vulnerability to hypertension.

A significant difference in hypertension prevalence was also observed across age groups, with 48.5% of individuals aged 40 years and above being hypertensive, compared to 16.36% in those under 40 consistence with earlier reports^16^, ^22^. This variation could be attributed to age-related physiological, hormonal, and psychosocial changes. These findings align with a study conducted among adults aged 35 to 74 in several low- and middle-income countries (LMICs), which reported prevalence rates of 49.9% in Kenya, 54.9% in South Africa, 52.5% in China, 32.5% in India, 42.3% in Pakistan, 45.4% in Argentina, 39.9% in Chile, 19.2% in Peru, and 44.1% in Uruguay 23. Hypertension was also significantly more prevalent among current smokers (55.17%) compared to non-smokers (32.14%) in conformity with previous reports 24, though contrary to some studies in Indonesia and South Korea which found no significant link between tobacco use and hypertension 25. This may be explained by the vasoconstrictive effects of tobacco chemicals, which narrow blood vessels over time. Similar results were reported in a study in China involving 8,801 participants, where 77.8% of current smokers had hypertension 26.

Educational level also showed an association with hypertension prevalence. Those with only primary education or no formal schooling had a prevalence rate of 36.59%, while individuals with secondary education or higher had a lower rate of 24.36%. This difference is likely due to limited awareness about hypertension risk factors among individuals with lower literacy levels. Comparable trends were observed in Colombia, where individuals with the lowest educational attainment had a 25% higher prevalence 15, and in Mongolia, where hypertension was more prevalent among less educated populations: 41.53% among Han and 48.08% among Mongolians with low education, compared to 17.75% and 23.11%, respectively, among the highly educated 27.

Income level, based on the World Bank classifications (as of July 1, 2023 – June 30, 2024), also influenced hypertension rates. Participants with low-middle income and above showed a prevalence of 47.37%, compared to 33.24% among those in extreme poverty or low-income groups. This may be due to higher stress levels and increased responsibilities among the more financially stable, possibly from supporting extended family members. These findings are consistent with a systematic review and meta-analysis in LMICs, which showed regional variations in hypertension prevalence between 1990 and 2020, including higher rates in South Asia (7.5%) and sub-Saharan Africa (6.04%), but a decline in Europe and Central Asia (-6.04%)^28^.

Marital status was another contributing factor. Married participants had a higher prevalence (37.56%) compared to those who were single (28.47%). This difference may be linked to ongoing socio-economic stressors. Similar trends were reported in Bhutan, where 45.4% of married men and 46.3% of married women had hypertension, compared to 35.1% and 14.1%, respectively, among those who had never married 12.

In exploring risk perception through five thematic areas: - causes, prevention and control, antihypertensive side effects, complications, and treatment adherence were adopted. The study found that each area influenced the likelihood of effective hypertension management. In line with the HBM, perceived severity played a crucial role in prompting individuals to take action. A Tanzanian study using the HBM similarly found that perceived severity was significantly associated with cues to action^25^. In this study, many participants identified persistent stress as a key contributor to hypertension, a finding consistent with a meta-analysis linking psychological stress to increased hypertension risk 22. Global urbanization, sedentary lifestyles, workplace stress, and lack of social support exacerbate anxiety and emotional strain, further increasing hypertension risk^19^.

Despite the overall good knowledge of risk factors like high salt, sugar, and fat intake, participants admitted that much of this awareness was gained after their diagnosis. This delayed knowledge acquisition is a concern. Prior research has established that high sodium intake raises blood pressure by promoting water retention, increasing systemic resistance, and affecting endothelial function and sympathetic activity 21.

In terms of prevention and control, participants understood stress management strategies such as spiritual intervention. However, some expressed difficulty in avoiding stress, particularly when root causes remain unresolved. This highlights the gap between theoretical knowledge and practical application. A qualitative study in South Bronx, New York, similarly reported that daily life stressors impeded effective hypertension control among African-American communities 29.

Regarding treatment adherence, side effects such as headaches and numbness were commonly reported and could lead to discontinuation. Nonetheless, many participants maintained good adherence, especially when they did not experience adverse effects. This supports findings from Sweden, where patients feared side effects but had limited understanding of hypertension and medication^30^.

Fear of complications such as stroke, numbness, and vision problems served as a strong motivator for adherence. In Iran, higher perceived risk of hypertension complications was significantly associated with better adherence to antihypertensive therapy 12.

A minority of participants believed hypertension was caused by witchcraft, with such beliefs often stemming from religious figures, thus influencing treatment decisions. Similar misconceptions have been reported in Ghana 31, Nigeria 32, and South Africa, underscoring the need for culturally sensitive education and intervention strategies. Misdiagnosis based on these beliefs can lead to incorrect disease management.

Although participants demonstrated a good understanding of hypertension risk factors: - such as excessive intake of salt, sugar, and fats/oils, they reported that this knowledge was mostly acquired after being diagnosed. This suggests that delayed awareness remains a critical issue among the study population. These findings align with previous research showing that high sodium intake contributes to elevated blood pressure through mechanisms like fluid retention, increased peripheral resistance, endothelial dysfunction, and changes in sympathetic nervous system activity 33.

In terms of prevention and control, many participants were aware of strategies to manage hypertension, such as adopting stress management techniques, including spiritual practices and efforts to reduce stress. However, some noted that stress was difficult to avoid, especially when its underlying causes were unresolved. This highlights a gap between theoretical knowledge and real-world feasibility. These observations are consistent with a qualitative study among African-Americans in the South Bronx, New York, which found that daily life stressors significantly hindered efforts to prevent and control hypertension 29.

Participants also reported experiencing side effects from antihypertensive medications, such as headaches and numbness, which sometimes led to treatment discontinuation. Nonetheless, most participants adhered to their treatment regimens, particularly when they did not experience adverse effects, resulting in better blood pressure control. These findings echo a qualitative study conducted in Sweden, which revealed that many patients had limited understanding of their condition and feared potential side effects from medication, influencing adherence^19^. Furthermore, complications such as stroke, numbness, and blurred vision were identified as major concerns among participants. The fear of developing such complications often motivated individuals to adhere to their prescribed medications. This is supported by a cross-sectional study from Iran, which found that a higher perceived risk of hypertension-related complications was significantly linked to better adherence to antihypertensive treatment^34^.

## Limitation of the study

The blood pressure results were based on one visit. Children below 18 years missed and their variables were not considered in the study yet some might have been victims of HTN. The income status of participant was assessed based on expenditure and hard cash earned by participants leaving out their assets or property owned by participants.

## Conclusions and recommendations

The study revealed the prevalence of 33.97% of hypertension among adults in Bududa town council, Bududa district, Uganda. The risk factors that were associated with hypertension included, age, and daily smokers in the past, stopped drinking due to health reasons, consumption of much salt and no MVPA due to walking or fitness. The risk perceptions that were associated with hypertension were, inappropriate source of information on the risk factors to hypertension, lack of knowledge on primary prevention, antihypertensive side effects, complication of hypertension like stroke and poor adherence to antihypertensive.

Therefore, government and other key stakeholder should direct their efforts towards promoting primordial and primary prevention rather than secondary and tertiary as it has been the case in Bududa town council. The emphasis should be directed towards health education on primary prevention, sensitization of the community on consumption of health food, family planning and engagement in socio-economic projects.

## Data Availability

All data generated or analyzed during this study are included in this published article and as supplementary materials

## List of abbreviations

HTN: hypertension
SBP: systolic blood pressure
DBP: diastolic blood pressure
SSA: Sub-Sarahan Africa
HIMS: Health management information system
WHO: World Health Organization
NGO: Non-government organizations
BMI: Body mass index
HBM: Health Belief Model
NCD: Non-communicable diseases
